# Concordance study of bronchial biopsy tissues and matched bronchial brushing cytology specimens in driver gene detection of non-small cell lung cancer

**DOI:** 10.3389/fonc.2026.1842742

**Published:** 2026-06-29

**Authors:** Xiongfeng Li, Lexia Chen, Peijia Niu, Jing Wang, Xupeng Sun, Ning Lu, Yahan Weng

**Affiliations:** Department of Pathology, Fuwai Central China Cardiovascular Hospital, Zhengzhou, China

**Keywords:** bronchial biopsy tissue, bronchial brushing cytology specimen, concordance, gene detection, non-small cell lung cancer (NSCLC)

## Abstract

**Objective:**

To clarify the correlation between clinicopathological features and gene mutation status in bronchial biopsy tissues and matched bronchial brushing cytology specimens from patients with non-small cell lung cancer (NSCLC), with a focus on verifying the concordance of driver gene detection between the two types of specimens, so as to provide high-quality clinical evidence for specimen selection in gene testing for NSCLC targeted therapy.

**Methods:**

A total of 160 bronchial biopsy tissue specimens from NSCLC patients pathologically diagnosed in our hospital from January 2023 to December 2025 were enrolled, among which 120 paired qualified bronchial brushing cytology specimens were included as the research objects. Amplification refractory mutation system polymerase chain reaction (ARMS-PCR) was performed to detect common driver genes of NSCLC in the two types of specimens. Clinicopathological data including gender, age, smoking history, lymph node metastasis status and pathological type were collected. SPSS 26.0 software was used for corresponding statistical analysis.

**Results:**

Among the 160 bronchial biopsy tissue specimens, the percentage of gene mutations/fusions was 63.75% (102/160). Among the 120 biopsy tissue specimens with paired bronchial brushing cytology samples, the percentage was 68.33% (82/120), while the percentage for the 120 paired bronchial brushing cytological specimens was 75.00% (90/120); In the two types of paired specimens, the percentages of gene mutations/fusions in female patients and lung adenocarcinoma patients were significantly higher than those in male patients and non-adenocarcinoma patients (P<0.05), whereas age, lymph node metastasis status, and smoking history showed no significant correlation with gene mutation status (P>0.05). The mutation profiles of both paired specimen types were predominantly epidermal growth factor receptor (EGFR) mutations, with EGFR mutation rates detected in 120 biopsy tissue samples at 43.33% and in cytological specimens at 50.83%.The overall concordance rate of genetic testing results between the two types of paired specimens reached 91.67%. The consistency test showed a Kappa value of 0.685 (P < 0.05), indicating a high degree of statistical consistency between the test results of the two specimen types.

**Conclusion:**

The gene mutation status of NSCLC patients is closely correlated with gender and pathological type.Paired bronchial brushing cytology specimens and bronchial biopsy tissue specimens collected synchronously exhibit high concordance in detecting NSCLC driver genes,and can be used as an effective supplementary specimen to bronchial biopsy tissues. It provides a new approach for gene detection in NSCLC patients who cannot tolerate invasive biopsy or have unqualified biopsy specimens, and facilitates the implementation of clinical precise targeted therapy.

## 1 Introduction

Lung cancer, as one of the malignancies with the highest incidence and mortality rates worldwide, NSCLC accounting for 80%-85% of total lung cancer cases, making its precise diagnosis and treatment a key research focus in the global oncology field (1). With the deep integration of molecular oncology and precision medicine, targeted therapy based on driver gene mutations has become the first-line standard treatment strategy for advanced NSCLC.Precise driver gene testing serves as the core prerequisite for screening beneficiary populations and optimizing individualized diagnosis and treatment plans, with its detection efficacy closely related to patients’ prognostic outcomes. Bronchoscopic tissue biopsy specimens represent the traditional “gold standard” for NSCLC driver gene testing; however, this technique has unavoidable clinical limitations. First, due to its invasive nature, the procedure is contraindicated for patients with severe underlying diseases, advanced age, physical frailty, or coagulation dysfunction, making it impossible for some patients to complete sample collection. Secondly, due to the heterogeneity of the tumor. single-site tissue biopsy struggles to comprehensively reflect the overall molecular phenotype of the tumor, often leading to false-negative results.Thirdly, biopsy specimens frequently encounter issues such as insufficient tissue volume or tumor cell content falling below the detection threshold (typically≥20%), with clinical specimen rejection rates as high as 15%-30%. Consequently, some patients miss the window for targeted therapy. Therefore, exploring safe, minimally invasive, and efficient alternative or supplementary testing specimens has become a critical scientific issue urgently needing resolution in the field of precision diagnosis and treatment for NSCLC.

Bronchial brushing cytology specimens, as minimally invasive samples obtained simultaneously with bronchoscopic biopsy, offer advantages such as simple operation, minimal trauma, capability for multi-site sampling, and good repeatability. Moreover, their sampling range can cover bronchial mucosal areas difficult to access by biopsy, theoretically compensating for the technical limitations of tissue biopsy. In recent years,the improved sensitivity of molecular detection techniques has created conditions for the clinical application of cytological specimens. However, existing research still exhibits academic gaps. The NSCLC driver gene spectrum shows significant racial disparities(the EGFR mutation rate in Asian populations reaches 30%–50%, higher than that in Western populations). Most previous consistency studies have been based on Western populations, while large-sample, multi-gene paired validation data for Chinese populations remain relatively scarce (2).The core obstacle to the widespread application of this technology lies in the suboptimal consistency of previous research findings. Most existing literature is based on traditional direct smear techniques, where brush biopsy specimens are fixed in 95% ethanol for preservation. Such specimens often suffer from delayed fixation or exposure to desiccation, leading to DNA degradation, high cell loss rates, and significant nucleic acid degradation, thereby affecting detection sensitivity. This has led the academic community to remain skeptical about whether cytological specimens can replace tissues, and no consensus has yet been reached on their clinical applicability and standardized procedures. This study employs ThinPrep cell preservation solution to standardize liquid-based cytological preservation for brush biopsy specimens. The fixative rapidly inactivates nucleases, thereby preventing DNA degradation and effectively circumventing issues of cellular desiccation and nucleic acid degradation inherent in conventional slide preparation processes. This approach significantly enhances the integrity and purity of DNA. Based on this, the study included a corresponding number of specimens, employed ARMS-PCR technology to detect driver genes, analyzed the influencing factors of gene mutations, and verified the consistency between brush cytology specimens prepared using this method and biopsy tissues in the detection of common driver genes. It clarified the clinical application value of bronchial brush cytology specimens in NSCLC gene testing, which can serve as a reliable alternative for NSCLC patients unable to obtain sufficient biopsy tissue. This provides evidence-based support for specimen selection in gene testing, expands the specimen sources for NSCLC gene detection, and enables more patients to benefit from precise targeted therapy.

## 2 Materials and methods

### 2.1 Study subjects

Patients who underwent bronchoscopy with synchronous collection of cytology and biopsy tissue specimens from January 2023 to December 2025 were selected. Bronchial biopsy tissue specimens from inpatients sent to the pathology department were screened according to the following criteria: biopsy tissue specimens pathologically diagnosed as NSCLC by conventional hematoxylin-eosin (HE) staining and immunohistochemical staining. Cases with biopsy tissue size less than 0.3 cm and tumor cell proportion lower than 20% were excluded, and a total of 170 valid specimens were finally obtained. Meanwhile, the cytology specimens corresponding to these 170 biopsy tissues were screened. Cases with only one cell brush slide retained, high proportion of necrotic tissue or inflammatory cells in the cell brush slide, and tumor cell proportion lower than 20% were excluded. Finally, 128 cases with valid and qualified corresponding cell brush slides were obtained. After obtaining ethical approval from the hospital ethics committee, we communicated with the patients of the selected specimens, informed them of the purpose of specimen use and related matters, and finally obtained informed consent from 160 patients with biopsy tissues and 120 patients with cytology specimens, who signed electronic informed consent forms. This study followed the ethical principles of the Declaration of Helsinki. Relevant data of the included patients were collected, including gender, age, smoking history, lymph node metastasis status, and pathological diagnosis type.

### 2.2 Specimen collection

The paraffin blocks corresponding to the 160 biopsy tissue specimens were selected from the pathological paraffin block archive according to the pathological numbers. Then, an unstained section with a thickness of 3 μm was cut from each block, re-stained with conventional HE, and re-diagnosed as NSCLC by a diagnostic pathologist. Using a Leica rotary microtome dedicated to molecular detection, 10–12 paraffin rolls were cut from each specimen and placed in a 1.5 ml enzyme-free centrifuge tube (EP tube) for subsequent gene detection. (When cutting different tissues each time, the blade was replaced, and the knife holder was wiped in the same direction with a clean cotton swab soaked in 100% ethanol to prevent cross-contamination).

**Figure 1:**
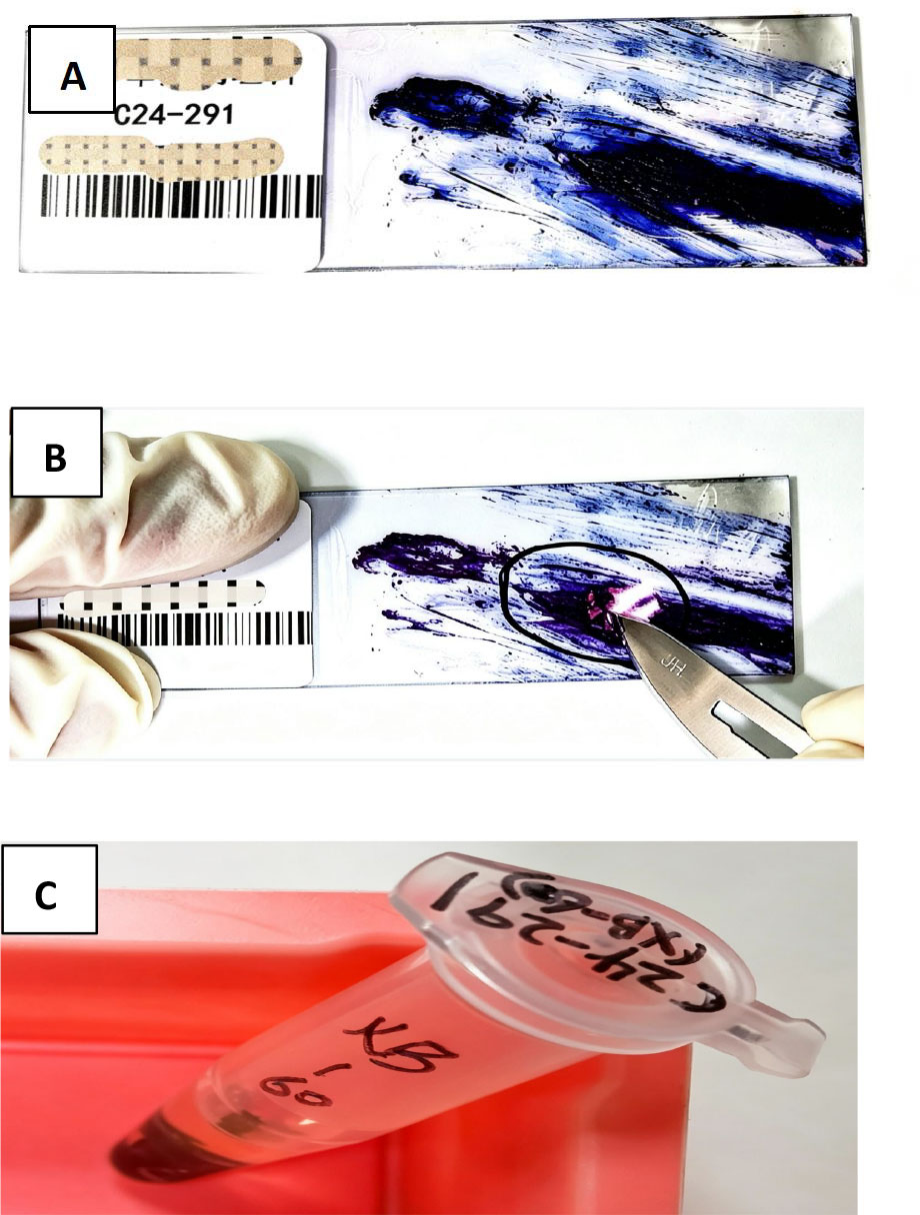
The process of cell specimen collection. **(A)** Selection of qualified cytological specimens **(B)** Delineation of the tumor area during follow-up and scraping of cancer cells **(C)** Collection of cancer cells in EP tubes for subsequent use.

The cell brush slides corresponding to the 120 cytology specimens were selected from the pathological cell archive according to the pathological numbers. A diagnostic pathologist re-examined the slides to confirm the presence of cancer cells, and marked the tumor cell area on the back of the slide with a marker pen. The cytology slides were placed in clean xylene for stripping for 10–30 minutes, the cover slips were gently removed, and the samples were treated with gradient alcohol (100%, 95%, 85%) for 1 min each, rinsed with tap water for 2 min, and dried at room temperature. Subsequently, a disposable blade was used to scrape the tumor cells, and the effective components were placed into the corresponding EP tubes (**Figure 1**) for subsequent gene detection.

### 2.3 Nucleic acid extraction and quality control

FFPE DNA/RNA Extraction Kit (Amoy Diagnostics, Xiamen, China) was used to extract nucleic acid from the two types of specimens respectively, with an elution volume set to 80 μL. The concentration of DNA and RNA was determined by NanoDrop 2000. The qualified DNA concentration was higher than 2 ng/μL, the RNA concentration was within the range of 10–500 ng/μL, and the OD_260_/OD_280_ ratio of both DNA and RNA was between 1.8 and 2.1.

### 2.4 Gene detection method

ARMS-PCR was performed using 5 mutation gene detection kits (Amoy Diagnostics, Xiamen, China), and amplification was carried out on the Roche cobas z 480 system.

### 2.5 Statistical methods

SPSS 26.0 software was used for statistical analysis. Count data were expressed as number (percentage) [n (%)], and the chi-square (χ²) test was used for comparison between groups. Cohen’s Kappa coefficient was used for concordance analysis, and the degree of concordance was interpreted according to the standard criteria for Kappa value. P-value<0.05 was considered statistically significant. Given the slight difference in age distribution between the full cohort and the paired subset, age grouping was performed using the median age specific to each respective cohort to ensure balanced comparison.

## 3 Results

### 3.1 Association between clinicopathological characteristics of biopsy tissues and gene mutation status

The genetic testing results of biopsy tissues from 160 NSCLC patients showed that the percentage of cases with mutations/fusions was 63.75% (102/160). The percentages across clinical subgroups were as follows: males 50.00% (45/90), females 81.43% (57/70); the patient age range was 21–85 years, with a median age of 64 years (IQR range: 56.0–69.2 years), with 58.44% (45/77) mutation-positive in subjects >64 years and 68.67% (57/83) in subjects ≤64 years. Mutation is found in 65.57% (40/61) cases with lymph node metastases, and 62.63% (62/99) cases without lymph node metastases. Mutation is found in 53.97% (34/63) cases with a smoking history, and 70.10% (68/97) cases without a smoking history. For lung adenocarcinoma, mutation is found in 77.17%(98/127) cases and 12.12%(4/33) of non-adenocarcinoma cases. Correlation analysis showed that mutation status was significantly associated with gender (P = 0.0001) and pathological type (P<0.0001), but not with age (P = 0.2377), lymph node metastasis status (P = 0.8357), or smoking history (P = 0.0567) (**Table 1**).

**Table 1:** Association between clinicopathological characteristics and gene mutation status in 160 biopsy tissue samples.

Clinical pathological features	Variable	Number of cases(160)	Positive rate	Chi square (X^2^)	P value
Sex	Male	90	45 (50.00%)	15.4974	0.0001
	Female	70	57 (81.43%)		
Age	>64	77	45 (58.44%)	1.3943	0.2377
	≤64	83	57 (68.67%)		
Lymph node metastasis status	Present	61	40 (65.57%)	0.043	0.8357
	Absent	99	62 (62.63%)		
Smoking status	Former or current	63	34 (53.97%)	3.6328	0.0567
	Never	97	68 (70.10%)		
Pathological type	Adenocarcinoma	127	98 (77.17%)	45.1808	<0.0001
	Other types other than adenocarcinoma	33	4 (12.12%)		

Among the 120 paired bronchial brushing cytology specimens and corresponding biopsy tissues, the percentage of cases with mutations/fusions was 68.33% (82/120). The percentages across clinical subgroups were as follows: males 60.32% (38/63), females 77.19% (44/57); patient age range was 21–85 years, with a median age of 61.8 years (IQR range: 56.0–68.3 years), with 62.90% (39/62) mutation-positive in subjects >61.8 years and 74.14% (43/58) in subjects ≤61.8 years. Mutation is found in 69.57% (32/46) cases with lymph node metastases, and 67.57% (50/74) cases without lymph node metastases. Mutation is found in 61.54% (24/39) cases with a smoking history, and 71.60% (58/81) cases without a smoking history. For lung adenocarcinoma, mutation is found in 77.88% (81/104) cases and 6.25% (1/16) of non-adenocarcinoma cases. Correlation analysis showed that mutation status was significantly associated with gender (P = 0.0472) and pathological type (P<0.001), but not with age (P = 0.1856), lymph node metastasis status (P = 0.8192), or smoking history (P = 0.2669) (**Table 2**).

**Table 2:** Association between clinicopathological characteristics and gene mutation status in 120 paired biopsy tissue specimens.

Clinical pathological features	Variable	Number of cases(120)	Positive rate	Chi square (X^2^)	P value
Sex	Male	63	38 (60.32%)	3.938	0.0472
	Female	57	44 (77.19%)		
Age	>61.8	62	39 (62.90%)	1.7515	0.1856
	≤61.8	58	43 (74.14%)		
Lymph node metastasis status	Present	46	32 (69.57%)	0.0523	0.8192
	Absent	74	50 (67.57%)		
Smoking status	Former or current	39	24 (61.54%)	1.2328	0.2669
	Never	81	58 (71.60%)		
Pathological type	Adenocarcinoma	104	81 (77.88%)	32.88	<0.001
	Other types other than adenocarcinoma	16	1 (6.25%)		

### 3.2 Association between clinicopathological characteristics of cytological specimens and gene mutation status

Genetic testing of 120 bronchial brushing cytology specimens revealed that the percentage of cases with mutations/fusions was 75.00% (90/120). The percentages in each clinical subgroup were as follows: males 65.08% (41/63), females 85.96% (49/57); with 70.97% (44/62) mutation-positive in subjects >61.8 years and 79.31% (46/58) in subjects ≤61.8 years. Mutation is found in 76.09% (35/46) cases with lymph node metastases, and 74.32% (55/74) cases without lymph node metastases. Mutation is found in 71.79% (28/39) cases with a smoking history, and 76.54% (62/81) cases without a smoking history. For lung adenocarcinoma, mutation is found in 84.62% (88/104) cases and 12.50% (2/16) of non-adenocarcinoma cases. Correlation analysis showed that mutation status was significantly associated with gender (P = 0.0152) and pathological type (P<0.0001), but not with age (P = 0.3988), lymph node metastasis status (P = 1.0000), or smoking history (P = 0.7357) (**Table 3**).

**Table 3:** Association between clinicopathological characteristics and gene mutation status in 120 cytological specimens.

Clinical pathological features	Variable	Number of cases(120)	Positive rate	Chi square (X^2^)	P value
Sex	Male	63	41 (65.08%)	5.8925	0.0152
	Female	57	49 (85.96%)		
Age	>61.8	62	44 (70.97%)	0.7119	0.3988
	≤61.8	58	46 (79.31%)		
Lymph node metastasis status	Present	46	35 (76.09%)	0.0000	1.0000
	Absent	74	55 (74.32%)		
Smoking status	Former or current	39	28 (71.79%)	0.114	0.7357
	Never	81	62 (76.54%)		
Pathological type	Adenocarcinoma	104	88 (84.62%)	34.7115	<0.0001
	Other types other than adenocarcinoma	16	2 (12.50%)		

### 3.3 Biopsy tissue gene mutation/fusion profile

**Figure 2:**
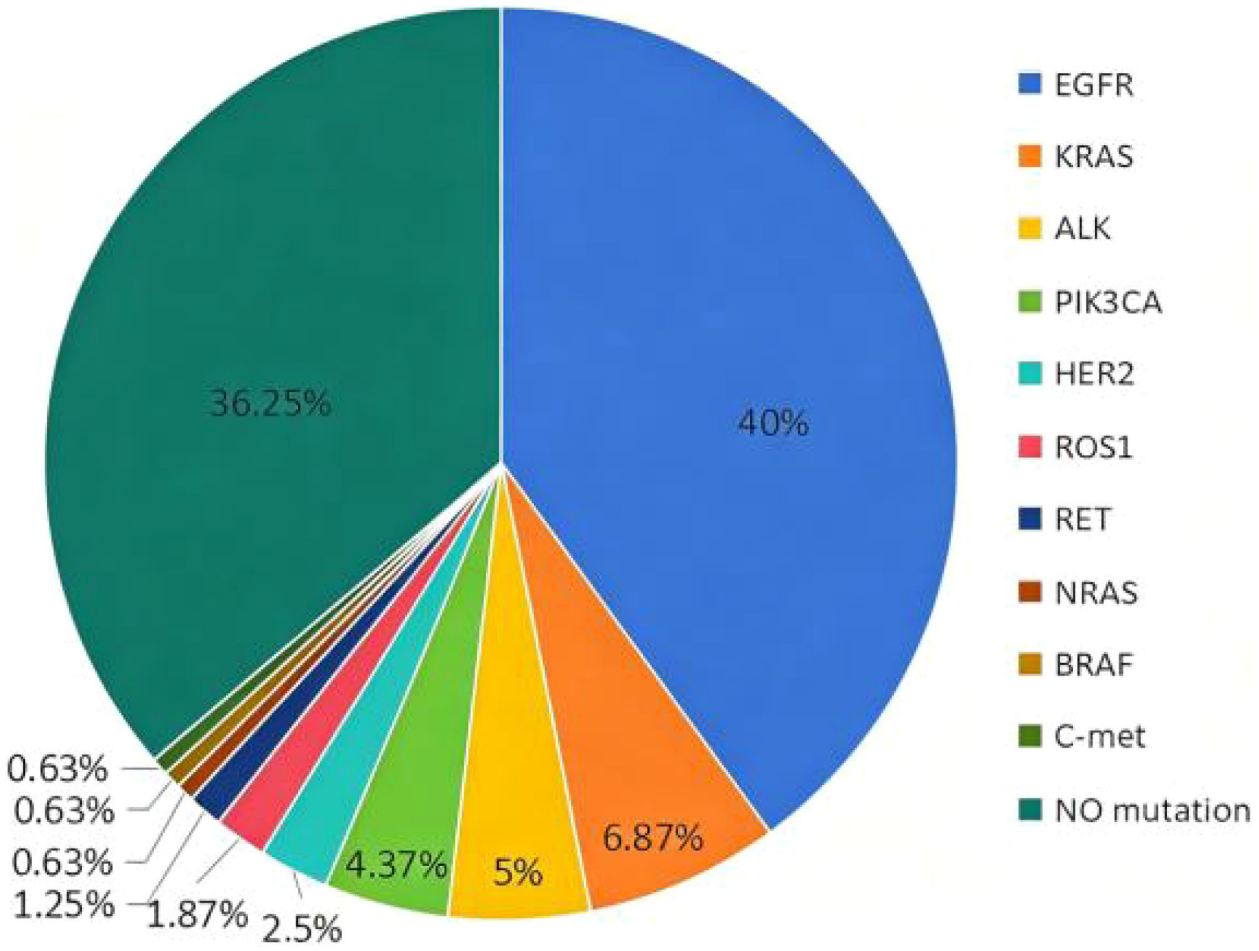
Gene mutation/fusion profiles of 160 biopsy tissues.

Among the 160 biopsy tissue specimens, the mutation percentages of EGFR, KRAS, PIK3CA, and HER2 were 40.00% (64/160), 6.87% (11/160), 4.37% (7/160), and 2.50% (4/160), respectively. The fusion percentages of ALK, ROS1, and RET were 5.00% (8/160), 1.87% (3/160), and 1.25% (2/160), respectively. The mutation percentages of NRAS, BRAF, and C-met were all 0.63% (1/160), while cases with negative genetic testing accounted for 36.25% (58/160) (**Figure 2**).

**Figure 3:**
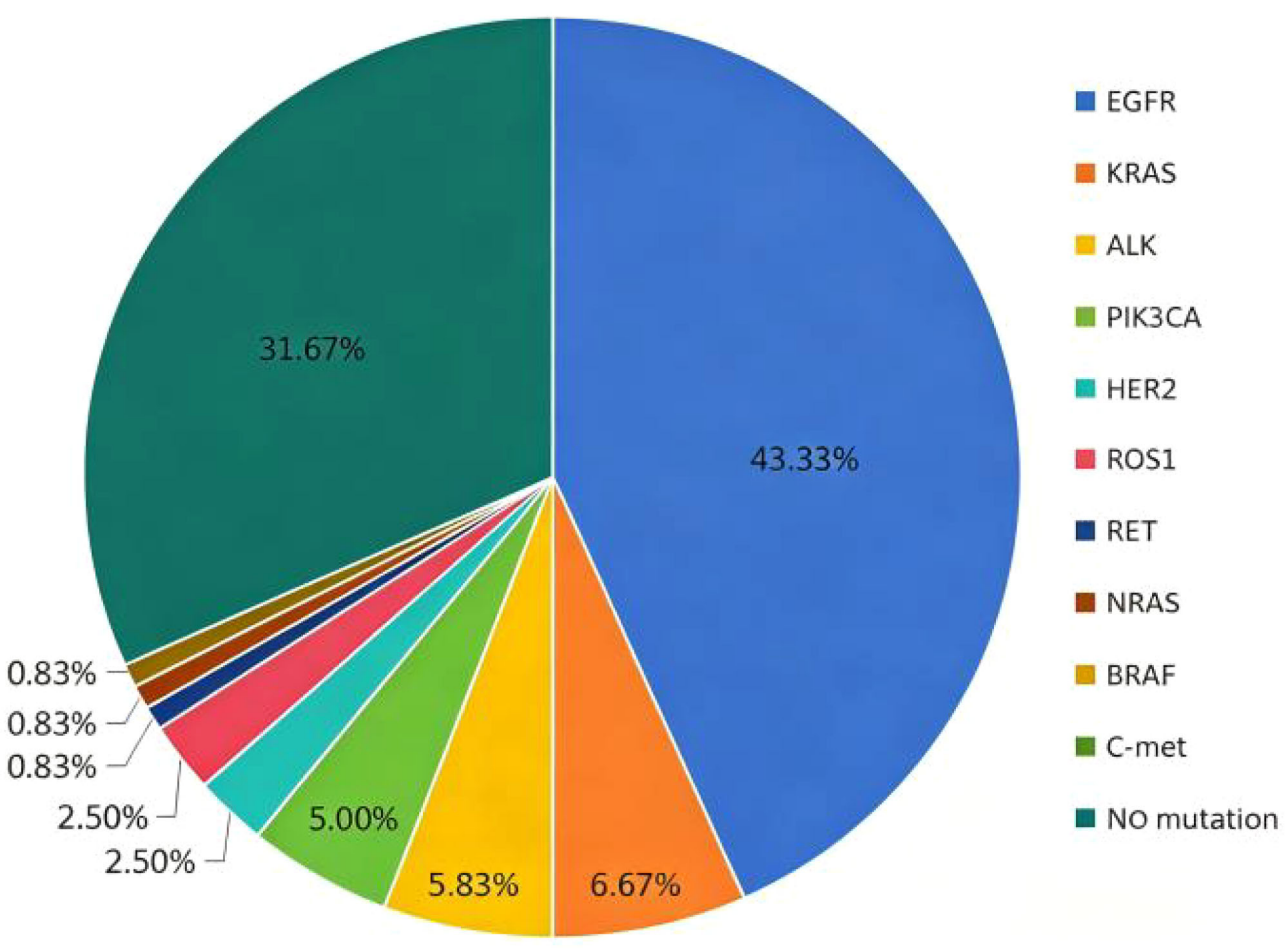
Gene mutation/fusion profiles of 120 paired biopsy tissues.

Among the 120 paired biopsy tissues, the mutation percentages of EGFR, KRAS, PIK3CA, and HER2 were 43.33% (52/120), 6.67% (8/120), 5.00% (6/120), and 2.50% (3/120), respectively. The fusion percentages of ALK and ROS1 were 5.83% (7/120) and 2.50% (3/120), respectively. The mutation/fusion percentages of RET, NRAS, and BRAF were all 0.83% (1/120), with no C-met mutations detected. Cases with negative genetic testing results accounted for 31.67% (38/120) (**Figure 3**).

### 3.4 Genetic mutation/fusion profile of cytological specimens

**Figure 4:**
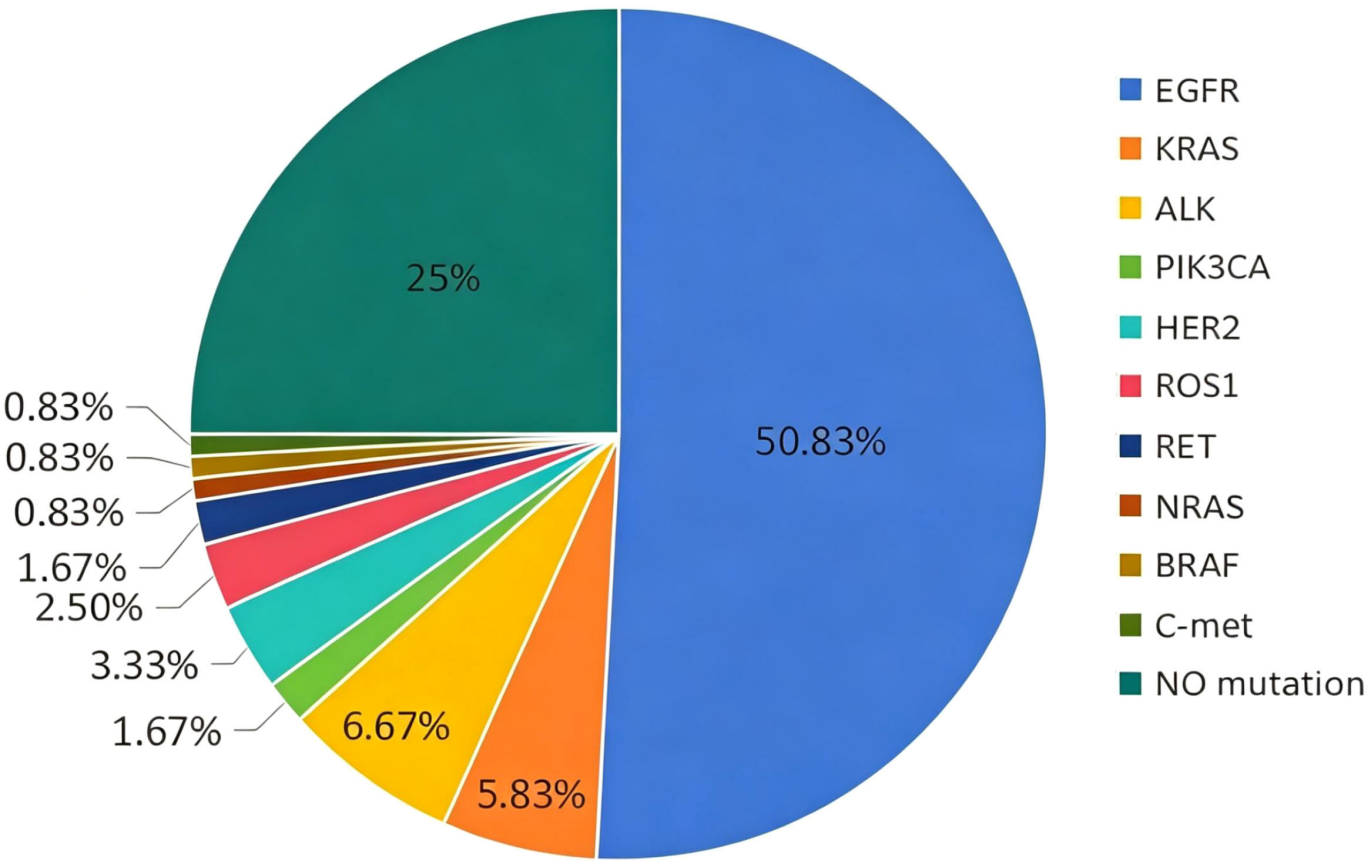
Gene mutation/fusion profiles of 120 cytological specimens.

Among the 120 cytological specimens, the mutation percentages of EGFR, KRAS, PIK3CA, and HER2 were 50.83% (61/120), 5.83% (7/120), 1.67% (2/120), and 3.33% (4/120), respectively; the fusion percentages of ALK, ROS1, and RET were 6.67% (8/120), 2.50% (3/120), and 1.67% (2/120), respectively; the mutation percentages of NRAS, BRAF, and C-met were all 0.83% (1/120), and cases with negative genetic testing accounted for 25.00% (30/120) (**Figure 4**).

### 3.5 Consistency of genetic testing results

Among the 120 paired specimens, the genetic test results of biopsy tissue specimens and cytological specimens were consistent in 110 cases, with a concordance rate of 91.67%. Using Cohen’s Kappa test method to evaluate the consistency of genetic testing between the two types of specimens, the results showed a Kappa value of 0.685 (P = 0.042). According to the interpretation criteria of the Kappa value, the two types of specimens achieved a high level of consistency in genetic testing. P < 0.05 indicates that this consistency is statistically significant (**Table 4**).

**Table 4:** Consistency analysis of gene detection positivity between biopsy tissues and cytological specimens.

Kappa consistency test				
Name	Kappa value	Standard error	Z price	P value
Biopsy tissue test positive Cytology specimen test positive	0.685	0.054	1.867	0.042

## 4 Discussion

This study confirms that gender and pathological type are key factors influencing the percentage of driver gene mutations/fusions in NSCLC. Among 160 biopsy tissues, the mutation/fusion percentage in female patients (81.43%) was significantly higher than that in males (50.00%). In 120 paired specimens, the percentage in biopsy tissues was 77.19% for females and 60.32% for males, while in cytological specimens, it was 85.96% for females and 65.08% for males. All differences exhibited statistical significance (P<0.05), and these findings align with the research conclusions of Ha SY et al. (3). The predictive value of pathological type for mutation status is extremely significant: in paired biopsy tissues, the mutation/fusion percentage in lung adenocarcinoma patients was as high as 77.88%, while in non-adenocarcinoma patients it was only 6.25%; in cytological specimens, the percentage for lung adenocarcinoma was 84.62%, compared to 12.50% for non-adenocarcinoma, with statistically significant differences between the two groups. These results align with the authoritative data in the “NCCN NSCLC 2024 Guidelines,” which states that “the cumulative incidence of targetable molecular alterations in lung squamous cell carcinoma is only 2%-10%” (4). Therefore, in clinical practice, a comprehensive gene testing strategy should be strictly implemented for lung adenocarcinoma patients, and non-adenocarcinoma NSCLC patients should not forgo testing, as some may still have the opportunity to receive targeted therapy (5, 6). In this study, the mutation/fusion percentages were slightly higher in patients without a smoking history compared to those with a smoking history, a finding consistent with research conclusions from East Asian populations (7). However, no statistically significant association was identified. It is speculated that the limited sample size in the subgroup of smoking patients may have hindered effective differentiation of the correlation between different mutation subtypes and smoking history, necessitating further expansion of the sample size for subsequent validation (7, 8). Additionally, no significant correlation was found between age, lymph node metastasis status, and driver gene mutations in both types of paired specimens. This indicates that driver gene mutations in NSCLC predominantly occur during the early stages of tumor development and serve as one of the initiating factors of tumorigenesis, with their status remaining unchanged as the disease progresses (9, 10).

In this study of 120 paired specimens, the EGFR mutation rate in biopsy tissues was 43.33%, while that in cytological specimens was 50.83%, representing a 7.5% increase compared to biopsy tissues. The overall driver gene-negative rate in cytological specimens was 25.00%, significantly lower than the 31.67% in biopsy tissues. This mutational profile data highly aligns with the epidemiological characteristics of the East Asian NSCLC population. The study by Liu H (11) indicates that the EGFR mutation percentage in East Asian NSCLC patients remains stable at 40%-50%; the multicenter study conducted by Lee B et al. (12), which included 6,595 patients, confirmed that the EGFR mutation percentage in the East Asian group was 42.5%, the KRAS mutation percentage was 6.7%, and the ALK fusion percentage was 5.8%. These findings are highly consistent with the results of paired biopsy tissues in this study, where KRAS was 6.67% and ALK fusion was 5.83%. Cytological specimens demonstrate superior detection efficacy with two primary underlying reasons: Firstly, the difference in specimen processing methods. Biopsy tissues fixed with formalin and embedded in paraffin (FFPE) are prone to DNA cross-linking and fragmentation. In contrast, this study employs ThinPrep liquid-based preservation solution, which can rapidly inactivate nucleases, thereby enhancing DNA integrity and purity. Secondly, the sampling method has advantages. The cell brush smear can perform brushing operations on multiple mucosal sites of the tumor lesion, enabling more comprehensive coverage of the qualitative regions of the tumor, thereby reducing the false negatives associated with single-point puncture biopsies (13, 14). The findings of this study supplement the epidemiological data on driver genes in the NSCLC population in central China, while confirming that cytological specimens can effectively detect core driver mutations, providing a foundation for precise diagnosis and treatment for patients from whom adequate tissue samples cannot be obtained (15).

This study confirmed through paired comparisons that the overall concordance rate between cytological specimens and biopsy tissue genetic testing reached 91.67%, with a Kappa value of 0.685, achieving a clinically recognized high level of agreement, which is consistent with the research findings of scholars such as Chua TH and Kim H (16, 17). In clinical practice, approximately 20%-30% of advanced NSCLC patients cannot obtain sufficient biopsy tissue due to factors such as tumor location and physical condition. Cytological specimens, with their advantages of being minimally invasive, safe, and repeatable, have become an important alternative specimen source (18–21). This study adopts the conventional clinical ARMS-PCR method, which aligns more closely with the practical conditions of primary hospitals in China. Cao Z et al. (22) confirmed that ARMS-PCR exhibits no significant difference in detection sensitivity and specificity between cytological specimens and biopsy tissues. Moreover, it is characterized by simple operation, short turnaround time, and low cost, making it suitable for nationwide promotion. Stringent pre-detection quality control is the key to reliable results: All cytological specimens included in this study were controlled to ensure tumor cell content of no less than 20%, with standardized nucleic acid concentration and purity quality control, effectively reducing the detection failure rate. This aligns with the research findings of Navani N et al. stating that “strict quality control can reduce the failure rate of cytological testing to below 5%” and Liu J et al. reporting that “when tumor cells account for ≥20%, the concordance rate between ARMS-PCR and tissue samples reaches over 90%” (23, 24). More importantly, the clinical value of cytological specimens is not limited to detection accuracy. Domestic real-world data show that the success rate of ARMS-PCR testing using bronchoscopic cytological specimens reaches 92.3%, with a targeted therapy matching rate of 48.7%. Multiple international studies have confirmed that targeted therapy guided by molecular subtyping based on cytological specimens yields progression-free survival (PFS) and objective response rates comparable to tissue-guided protocols, demonstrating clear prognostic value (17, 25–28).

This study employed a paired specimen design from the same patient, eliminating inter-patient tumor heterogeneity interference, significantly enhancing result reliability. Strict adherence to clinical routine testing procedures and quality control standards was maintained, utilizing the widely adopted ARMS-PCR method domestically. The research conclusions possess strong clinical applicability, systematically analyzing the correlation between clinicopathological characteristics and gene mutations/fusions, thereby providing reference for clinical specimen selection and testing indication determination. The study has the following limitations: ①The laboratory is not equipped with TapeStation instruments, making nucleic acid integrity assessment unfeasible; ②As a single-center retrospective study, the sample size was limited, particularly the undersized non-adenocarcinoma subgroup, which may affect the statistical power of subgroup analyses; ③Long-term patient follow-up was not conducted, resulting in a lack of survival outcome data for cytology-guided targeted therapy; ④Third-party validation was not performed on the 10 discordant specimens, Subsequent NGS-based analysis could elucidate the causes of discordance.

This study confirms that bronchial brushing cytology specimens and paired bronchial biopsy tissues exhibit high consistency in the detection results of core driver genes in NSCLC. Cytology specimens are minimally invasive, safe, and easily obtainable, and due to the advantages of liquid-based preservation and multi-site sampling, their efficacy in detecting driver genes surpasses that of conventional biopsy tissues. By strictly controlling the tumor cell proportion to no less than 20% and standardizing nucleic acid quality control, the reliability of ARMS-PCR detection results can be ensured. Therefore, bronchial brushing cytological specimens can serve as a reliable alternative for NSCLC patients who cannot obtain sufficient biopsy tissue, enabling driver gene testing and precise treatment decisions. In the future, with the advancement of molecular detection technologies, the combination of cytological specimens and NGS technology will further expand their application scenarios in the precision diagnosis and treatment of NSCLC, allowing more advanced patients to benefit from targeted therapy.

## Data Availability

The datasets presented in this study can be found in online repositories. The names of the repository/repositories and accession number(s) can be found in the article/supplementary material.
